# Improvement of Summer Green Tea Quality Through an Integrated Shaking and Piling Process

**DOI:** 10.3390/foods14071284

**Published:** 2025-04-07

**Authors:** Zheng Tu, Sixu Li, Anan Xu, Qinyan Yu, Yanyan Cao, Meng Tao, Shanshan Wang, Zhengquan Liu

**Affiliations:** 1 Institute of Sericulture and Tea, Zhejiang Academy of Agricultural Sciences, Hangzhou 310021, China; tuzheng@zaas.ac.cn (Z.T.); 15822005404@163.com (S.L.); grace@zaas.ac.cn (A.X.); 15329063264@163.com (Q.Y.); yycao@zju.edu.cn (Y.C.); teatmtm@163.com (M.T.); 7424817.kang@163.com (S.W.); 2 The College of Food and Health, Zhejiang A & F University, Hangzhou 311300, China

**Keywords:** summer green tea, shaking, piling, metabolomics, non-volatile metabolites

## Abstract

Summer green tea often suffers from an inferior flavor, attributed to its bitterness and astringency. In this study, an integrated shaking and piling process was performed to improve the flavor of summer green tea. The results demonstrated a significant improvement in the sweet and kokumi flavors, accompanied by a reduction in umami, astringency, and bitterness following the treatment. Additionally, the yellowness and color saturation were also enhanced by the treatment. A total of 146 non-volatile metabolites (NVMs) were identified during the study. The elevated levels of sweet-tasting amino acids (L-proline, L-glutamine, and L-threonine), soluble sugars, and peptides (such as gamma-Glu-Gln and glutathione) contributed to the enhanced sweetness and kokumi. Conversely, the decreased levels of ester-catechins, flavonoid glycosides, and procyanidins resulted in a reduction in umami, astringency, and bitterness. Furthermore, the decreased levels of certain NVMs, particularly ascorbic acid and saponarin, played a crucial role in enhancing the yellowness and color saturation of the summer green tea. Our findings offered a novel theoretical framework and practical guidelines for producing high-quality summer green tea.

## 1. Introduction

Green tea has garnered global recognition as a daily beverage due to its distinctive flavor profile and well-documented health benefits. Its unique processing techniques preserve a high content of bioactive components in the tea leaves, such as polyphenols, amino acids, and alkaloids [1]. These components exhibit multifunctional bioactivities, including antioxidant, anti-inflammatory, anticancer, hypoglycemic, and hypolipemic properties [2]. Furthermore, these non-volatile metabolites (NVMs) are closely related to the quality of green tea. Critically, NVMs directly determine tea quality: polyphenols govern astringency and infusion intensity [3], caffeine contributes to bitterness while imparting refreshing effects [4], and amino acids (notably L-theanine, L-glutamic acid, and aspartic acid) establish freshness and a mellow taste while participating in aroma formation [5]. Furthermore, amino acids exhibit flavor-enhancing synergies with other food components [6]. Sugars modulate sensory balance by mitigating astringency and enhancing sweetness [5].

Summer green tea has been characterized by excessive bitterness and astringency due to its high content of tea polyphenols, caffeine, and other compounds, resulting in inferior tea quality. This has led to a series of issues, including low production enthusiasm among tea farmers, reduced harvesting volumes, inefficient utilization of summer tea, and resource wastage [7]. The traditional processing methods for green tea primarily encompass spreading, fixing, rolling, and drying. Currently, numerous studies have been dedicated to improving traditional processing techniques in order to reduce the bitter and astringent taste of summer green tea. For instance, methods include extending the spreading duration, adopting electromagnetic roller–hot-air–steam triple-coupled fixation, and decreasing the temperatures for drying [8,9,10].

In addition, recent research has incorporated the shaking process to alleviate the bitter and astringent taste of summer green tea [11]. The shaking process involves rotating the tea leaves on a shaking machine, which primarily comes from oolong tea processing. Through water loss and mechanical damage, it promotes friction and injury to the leaf edges, stimulating the activity of enzymes such as oxidase, hydrolase, and reactive oxygen species metabolic enzymes, and enriching biosynthetic metabolism [12]. This shaking process can facilitate the oxidation of catechins and flavanol glycosides, accelerate the decarboxylation of glutamic acid, and subsequently promote the formation of theaflavin-3,3′-digallate, thereby enhancing the sweetness of the green tea [13]. On the other hand, humid-heat treatment can significantly reduce the bitter and astringent taste, as well as other unwanted flavors in the tea, playing a notable role in improving the taste quality of summer green tea [7]. Common humid-heat treatments include the piling process of yellow tea and dark tea [14,15]. During the piling process, esterified catechins are converted into simple catechins through hydrolysis under humid-heat conditions, contributing to the kokumi and mellow taste of teas [16]. As the piling time increases during the process, the water extractables in the tea leaves continue to rise, while the content of tea polyphenols gradually decreases [17].

In summarizing the relevant research on improving the quality of summer green tea, most existing studies have focused on the optimization of singular processing. However, critical gaps persist in understanding the synergistic effects of combined processing techniques on objective quality metrics, particularly given the biochemical complexity of NVMs governing tea flavor profiles. In this study, a non-targeted metabolomic analysis based on ultra-performance liquid chromatography–triple quadrupole time-of-flight mass spectrometry (UPLC-Triple-TOF-MS) was employed to reveal the effects of combining the shaking and piling methods on enhancing the flavor quality of summer green tea.

## 2. Materials and Methods

### 2.1. Materials and Instruments

Longjing 43 (*Camellia sinensis* L.) cultivars with three or four leaves were harvested in Lishui, Zhejiang, China, in July 2024.

The methanol and acetonitrile of high-performance liquid chromatography (HPLC) grade were obtained from Fisher Chemical (Loughborough, UK). Formic acid (HPLC grade) was purchased from CNW (Shanghai, China). Isopropanol (HPLC grade) was purchased from Merck (Darmstadt, Germany).

The UPLC-Triple-TOF-MS system was purchased from AB SCIEX (Darmstadt, Germany). The spectrophotometer (CM-5) used for colorimetric measurement was obtained from Konica Minolta Holdings, Inc. (Tokyo, Japan). The hot-air drying machine (6CHZ-9B) and shaking machine (6CWY-90) were purchased from Fujian Jiayou Tea Patternry Intelligent Technologies Inc. (Fujian, China). The rolling machine (6CR-40) and fixing machine (6CST-60) were acquired from Zhejiang Lvfeng Patternry Co., Ltd. (Zhejiang, China). The freeze dryer (Christ Beta 1-8 LSC BASIC) was purchased from Martin Christ Co., Ltd. (Osterode, Germany). The centrifuge (Multifuge X1 Pro) was purchased from Thermo Fisher Scientific (Waltham, MA, USA).

### 2.2. Sample Preparation

Four equal parts of fresh tea leaves were prepared for traditional green tea (GT), shaking-only green tea (SGT), piling-only green tea (PGT), and shaking and piling combined green tea (SPGT) processing. The specific processing methods were following our previously published article [18].

For the processing of GT, freshly plucked tea leaves were initially spread under controlled conditions of 27–33 °C and 45–55% relative humidity until achieving a 70% moisture content. Thermal fixation was subsequently performed using a rotary fixation machine at 260–280 °C. The softened leaves then underwent a 45 min rolling process comprising two distinct phases: initial pressure-free rolling (15 min) followed by compression rolling under heavy pressure (30 min). Primary drying was executed in a hot-air drying machine, reducing moisture content to 15–20%. After 30 min of ambient cooling, secondary drying at 90 °C for 30 min achieved final moisture levels of approximately 6%.

For the processing of SGT, a shaking process was added. To prevent excessive oxidation from causing the tea leaves to turn red, spread tea leaves were subjected to 1 min mechanical agitation in a shaking machine, followed by 60 min static oxidation prior to the fixation stage. The other processing steps and parameters were the same as the processing of GT.

For the processing of PGT, a piling process was added. To provide a suitable thermal-humid environment, rolled tea leaves were wrapped in wet gauze and piled in a circular box at 45 °C conditions before the drying step. The other processing steps and parameters were the same as the processing of GT.

For the processing of SPGT, the shaking and piling processes were combined with the processing steps and parameters of GT.

Process-intermediate samples from SPGT and representative finished products from all experimental groups were collected and lyophilized using a freeze dryer.

### 2.3. Sensory Evaluation and Colorimetric Measurement

A sensory panel of eight trained evaluators (3 males, 5 females; age range 24–45 years) conducted quantitative descriptive analysis of taste profiles for GT, SGT, PGT, and SPGT. Infusions were prepared in accordance with GB/T 23776-2018 [19], utilizing 3 g of tea leaves steeped in 150 mL of boiling deionized water for 5 min. Panelists evaluated the differential taste attributes (sweetness, bitterness, astringency, umami, and kokumi) using a 10-point scale: extremely weak (0–2 points), weak (2–4 points), neutral (4–6 points), strong (6–8 points), and extremely strong (8–10 points), following a previously reported method [20].

The colorimetric measurement was performed using a spectrophotometer. L* represents the brightness of tea infusions. The a* value at “−” represents the greenness of tea infusions, the b* value at “+” represents the yellowness of tea infusions, and the C* represents the color saturation of tea infusions. ΔE represents the color difference of tea infusions. The L*, a*, and b* values were detected directly by the spectrophotometer. C* and ΔE were further calculated according to the following formulas.

C* = [(a*)^2^ + (b*)^2^]^1/2^ (1)

ΔE = [(L*_sample_ − L*_GT_)^2^ + (a*_sample_ − a*_GT_)^2^ + (b*_sample_ − b*_GT_)^2^]^1/2^ (2)

### 2.4. Non-Targeted Metabolomics Analysis by UPLC-Triple-TOF-MS

Each tea sample was uniformly ground to powder. A total of 0.2 g of the tea powder was transferred to a 10 mL centrifuge tube. Then, 5 mL of 70% methanol (methanol/water, *v*/*v*) was added and immediately transferred to a 70 °C water bath for extraction for 10 min (stirring every 5 min). After it cooled to room temperature, the tube was centrifuged at 3500 rpm for 10 min in a centrifuge. Finally, the supernatant was transferred to a 10 mL volumetric flask. The extraction procedures were repeated three times. The extracts were combined and adjusted to a 10 mL volume and then filtered through a 0.22 μm membrane for use.

The chromatographic column used was a BEH C18 column (100 mm × 2.1 mm, 1.7 µm; Waters, Milford, CT, USA). Mobile phase A consisted of water containing 0.1% formic acid, while mobile phase B was a mixture of acetonitrile/isopropanol (1/1, *v*/*v*) containing 0.1% formic acid. The gradient elution program was as follows: 0–3 min, 0–20% B; 3–9 min, 20–60% B; 9–11 min, 60–100% B; 11–13.5 min, 100% B; 13.5–13.6 min, 100% B; 13.6–16 min, 0% B. The flow rate was set at 0.4 mL/min, the injection volume was 20 μL, and the column temperature was maintained at 40 °C.

The mass spectrometry signal acquisition for the samples was conducted using both positive and negative ion scanning modes. The electrospray capillary voltage, injection voltage, and collision voltage were set to 1.0 kV, 40 V, and 6 eV, respectively. The ion source and desolvation temperature were maintained at 120 °C and 500 °C, respectively. The carrier gas flow rate was 900 L/h, the mass spectrometry scanning range was 50–1000 *m*/*z*, and the resolution was 30,000.

### 2.5. Identification of Metabolites

Before conducting the statistical analysis, a series of preprocessing steps needed to be performed on the raw data. Firstly, a metabolomics processing software, Progenesis QI v3.0 (Waters Corporation, Milford, CT, USA), was used for baseline filtering, peak identification, integration, retention time correction, and peak alignment of the raw data. Subsequently, data preprocessing was carried out to obtain a final data matrix for subsequent analysis. The raw data were also searched and identified using the metabolomics processing software Progenesis QI by matching MS and MS/MS mass spectrometry information against metabolic databases. The primary databases used included http://www.hmdb.ca/ (accessed on 22 January 2025) and https://metlin.scripps.edu/ (accessed on 22 January 2025), as well as a self-constructed database.

### 2.6. Statistical Analysis

The NVMs were quantified using Microsoft Excel 2024 (Microsoft Corp., Redmond, WA, USA) for preliminary data organization. Data visualization was implemented through OriginPro 2022 (OriginLab Corporation, Northampton, MA, USA), generating radar plots and frequency distribution histograms. Multivariate statistical analyses, including principal component analysis (PCA), hierarchical clustering analysis (HCA), differential metabolite screening (volcano plots), heatmap profiling, and metabolite correlation networks, were executed via the Metware Cloud platform (https://cloud.metware.cn/ accessed on 22 January 2025). Statistical significance was determined by one-way analysis of variance (ANOVA) with Tukey’s post hoc test (α = 0.05), performed in SPSS Statistics 23.0 (IBM Corp., Armonk, NY, USA).

## 3. Results

### 3.1. Variations in Infusion Color and Taste Among GT, SGT, PGT, and SPGT

**Figure 1:**
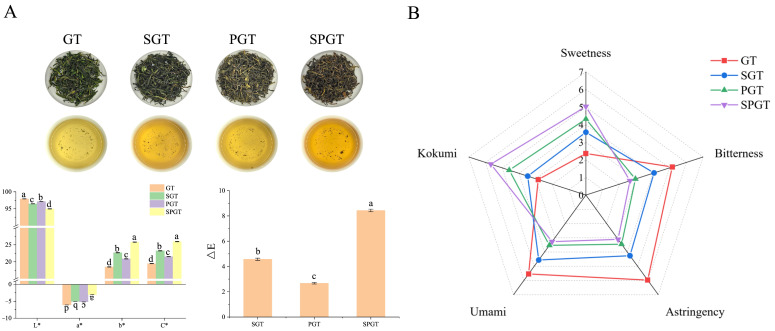
Infusion color and taste characteristics in GT, SGT, PGT, and SPGT. (**A**) The appearance and color differences. (**B**) Taste evaluation of differential taste characteristics. Different letters mean a significant difference at *p* < 0.05.

The treatments of shaking and piling showed a significant impact on the appearance and infusion color of summer green tea samples (Figure 1A). Compared with GT, the L* value of the green tea infusions processed with shaking (SGT) and piling (PGT) decreased significantly (*p* < 0.05), and significant changes also occurred in the a* value and b* value (*p* < 0.05). These results indicated that shaking and piling treatments reduced the brightness of the infusion color while increasing its yellowness. Additionally, the C* value of SGT and PGT increased significantly (*p* < 0.05), suggesting that shaking and piling treatments improved the color saturation of the tea infusion. The changes of SPGT showed these effects were significantly enhanced when shaking and piling treatments were combined (*p* < 0.05). Analysis of the ΔE results also confirmed the above conclusions. These modifications aligned with prior reports attributing color transformations to enzymatic pigment remodeling during shaking and piling treatments [21,22].

Figure 1B revealed significant differences in taste intensity among GT, SGT, PGT, and SPGT. SPGT had the highest sweetness and kokumi but the lowest umami, astringency, and bitterness. SGT and PGT showed similar trends to SPGT, indicating that shaking and piling enhanced sweetness and kokumi while reducing umami, astringency, and bitterness. These differences could be attributed to the stress caused by the shaking process, which promoted the conversion of NVMs responsible for astringency and bitterness, such as catechins, flavonoids, caffeine, etc. [13]. On the other hand, the humid-heat effect induced by piling, leading to the hydrolysis of proteins and starches, thereby facilitating an increase in the content of soluble sugars and free amino acids, also contributed to the formation of kokumi and sweetness tastes [17]. In summary, both shaking and piling have demonstrated significant improvement in the taste of summer green tea, and the effect was intensified when these two processes integrated.

### 3.2. Overall Determination of NVMs Among GT, SGT, PGT, and SPGT

**Figure 2:**
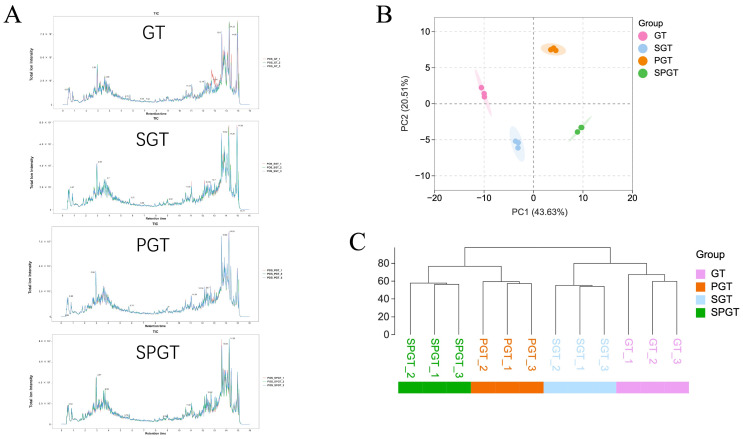
Metabolomics analysis of NVMs in GT, SGT, PGT, and SPGT. (**A**) Total ion chromatogram obtained with UPLC-Triple-TOF-MS. (**B**) PCA score plot. (**C**) Cluster analysis.

The flavor characteristics of green tea are governed by synergistic interactions among diverse NVMs, rather than individual compounds. In this study, we employed an integrated metabolomics and multivariate statistical approach to analyze NVM profiles across four tea variants: GT, SGT, PGT, and SPGT. Total ion chromatograms revealed distinct metabolite fingerprints among the samples (Figure 2A), with 146 NVMs identified, encompassing amino acids and derivatives, catechins, procyanidins, caffeine, flavonoids, organic acids (phenolic and condensed forms), tea pigments, peptides, nucleotides, and soluble sugars (Table S1). To further investigate the differences in NVMs among them, an unsupervised principal component analysis (PCA) was performed. As depicted in Figure 2B, the first two principal components explained 43.63% and 20.51% of the total variation, respectively, with a combined contribution of 64.14%. Four tea samples processed by different methods were distinctly grouped, indicating that shaking, piling, and their combination significantly affected the NVMs (*p* < 0.05). The cluster analysis supported these differences and indicated that SGT was closer to GT, while SPGT was clustered together with PGT in flavor profile (Figure 2C).

#### 3.2.1. Amino Acids and Derivatives

**Figure 3:**
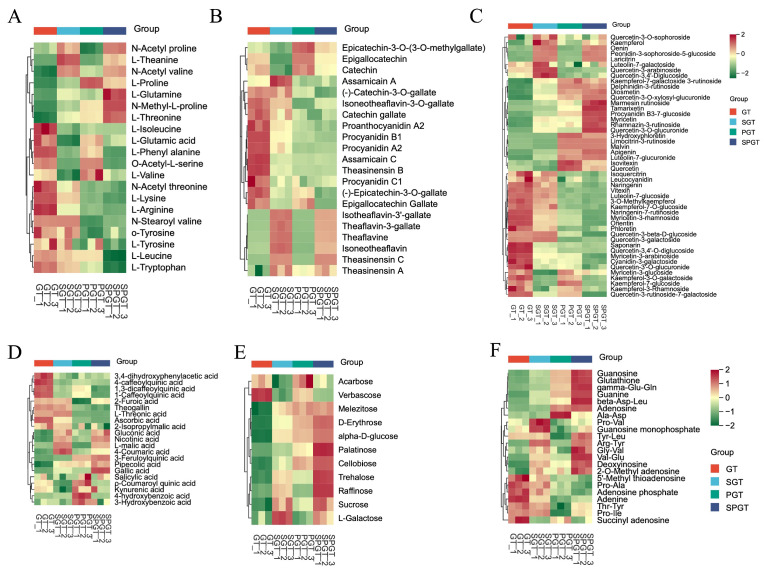
Heat maps of classified NVMs detected in GT, SGT, PGT, and SPGT. (**A**) Amino acids and their derivatives. (**B**) Catechins and tea pigments. (**C**) Flavonoids and their glycosides. (**D**) Organic acids. (**E**) Soluble sugars. (**F**) Peptides and nucleotides.

Amino acids play a pivotal role in shaping the mellow and refreshing taste profile of green tea, categorized by taste attributes into umami, bitter, sweet, and salty groups [3]. In this study, 20 amino acids were identified through metabolomic analysis, exhibiting distinct modulation patterns across processing methods (Figure 3A). Notably, L-theanine, renowned for its umami taste in high-quality green tea [6], showed increased levels in both SGT and SPGT. Sweet-tasting amino acids, like L-proline, L-glutamine, and L-threonine, increased in SGT, PGT, and SPGT. Conversely, bitter-tasting amino acids, such as L-isoleucine, L-phenylalanine, L-valine, L-lysine, L-arginine, and L-leucine, decreased in SPGT.

The mechanical stress inflicted by the shaking process triggered protein hydrolysis and dynamic alterations in endogenous metabolites, ultimately boosting the amino acid content [23]. Moreover, the shaking process accelerated the deamination and decarboxylation reactions of amino acids, paving the way for subsequent Strecker degradation and Maillard reactions and fostering an environment conducive to the development of the aroma characteristics of tea [24]. Adequate piling further enhanced the levels of amino acids through protein hydrolysis and the derivatization of specific amino acids. However, prolonged piling treatment could result in a significant decline in the levels of most amino acids [17].

#### 3.2.2. Catechins, Procyanidins, and Tea Pigments

Catechins, particularly ester-type catechins, constituted the primary contributors to the bitter and astringent taste of tea, comprising 12–24% of the dry weight of fresh tea leaves [25]. As illustrated in Figure 3B, the levels of ester-type catechins, such as epigallocatechin gallate, catechin gallate, (−)-epicatechin-3-O-gallate, and (−)-catechin-3-O-gallate, decreased in SPGT, suggesting that an integrated shaking and piling process effectively reduced bitter and astringent-related catechins. Similarly, procyanidins, which exhibited bitter taste characteristics, underwent a consistent reduction in SPGT, aligning with the transformation observed in ester-type catechins. Procyanidins, composed of structural units like (+)-catechin and (−)-epicatechin, might undergo degradation during the piling process [26,27]. Furthermore, the content of theaflavins, reddish-orange compounds biosynthesized from catechins, increased in SPGT. In addition, the elevated levels of theaflavins contributed to the consumption of catechins, thereby mitigating the bitterness and astringency of green tea.

Research has demonstrated that mechanical damage inflicted by shaking treatment stimulated an elevation in the activities of polyphenol oxidase and peroxidase, concurrently augmenting cell membrane permeability. This enhancement facilitated the interaction among catechins, ultimately resulting in a decline in catechin content and a corresponding surge in theaflavins [28]. Consequently, a notable increase in theaflavin content was evident in SGT. During the piling process, influenced by moisture and heat, partially oxidized catechins underwent isomerization and thermal cleavage, leading to a marked decrease in ester-type catechin content and their transformation into simpler catechins, such as epicatechin and epigallocatechin [17]. As a result, the accumulation of these simple catechins was observed in PGT. Furthermore, catechins, when oxidized by polyphenol oxidase, transformed into o-quinones, which, under conditions of high humidity and temperature, were prone to further oxidation, polymerization, and condensation, forming theaflavins [29]. However, the variation in theaflavin content in PGT was insignificant, suggesting that shaking treatment was the primary factor driving the increase in theaflavins.

#### 3.2.3. Flavonoids and Their Glycosides

Flavonoids were collectively referred to as a class of compounds possessing a 2-phenylchromone structure and readily undergo hydroxylation to produce flavonols. Both flavonoids and flavonols could easily combine with carbohydrates to form flavonoid glycosides and flavonol glycosides, respectively. Researchers have successively isolated flavonoids from tea, including monosaccharides, disaccharides, and trisaccharides of flavonols [25]. Flavonoid glycosides exhibited a velvety, soft astringency, playing a significant role in the presentation of the astringent taste in tea infusions [30]. As shown in Figure 3C, 19 flavonoids and 29 flavonoid glycosides were detected among four summer green tea samples. Although there was no consistent change, more than half of the flavonoid glycosides showed a decrease in SPGT, while most flavonoids exhibited an increase. This suggested that the combined process of shaking and piling could reduce the astringency caused by flavonoid glycosides. Observing the changes in SGT and PGT, it was evident that shaking and piling have different effects on different types of flavonoids and flavonoid glycosides. Compared to SGT, the contents of compounds such as malvin, apigenin, isovitexin, and quercetin were increased in PGT, while the contents of kaempferol, oenin, and laricitrin were notably decreased. This might be attributed to the different mechanisms of shaking and piling. After shaking treatment, dehydration stress and wounding stress stimulated the expression of flavonol glycoside-related synthetic enzymes (such as flavonol synthase and UDP-glucose flavonoid 3-O-glucosyltransferase genes). Simultaneously, the increased enzyme activity induced by shaking further promoted the oxidation of flavonol glycosides, while the inhibition of reactive oxygen species accumulation under stress also led to the degradation of flavonol glycosides [13]. Conversely, piling treatment facilitated the oxidation and degradation of flavonol glycosides through the combined effects of heat and moisture [14]. Overall, the combined treatment of shaking and piling has been proven to reduce the astringency caused by flavonoid glycosides.

#### 3.2.4. Organic Acids

Although most organic acids did not directly contribute to the taste of tea infusion, they played a crucial role as intermediate metabolites in the formation of tea flavor compounds [30,31]. A total of 21 organic acids were detected (Figure 3D), yet no consistent differences were observed among the tea samples. Notably, the content of gallic acid increased in SPGT. Gallic acid not only enhanced the positive taste of Japanese green tea but also served as a key factor influencing its sour and astringent taste [6,32]. This increase might be attributed to the process where shaking and piling facilitated the degradation of ester-catechins into gallic acid and simple catechins [14,33]. Furthermore, a decrease was observed in 1-caffeoylquinic acid, 4-caffeoylquinic acid, 3-feruloylquinic acid, ρ-coumaroylquinic acid, and 1,3-dicaffeoylquinic acid in SPGT, with similar changes also noted in SGT and PGT. Quinic acid not only contributed to the sour taste of tea but also enhanced its astringency [34]. These findings suggested that shaking and piling could improve the quality of summer green tea by reducing the content of several organic acids that impart a sour and astringent taste.

#### 3.2.5. Soluble Sugars

Soluble sugars were significant contributors to the sweetness of tea infusions, capable of mitigating their coarse and astringent flavors while enhancing their sweet and mellow taste [35]. Compared to GT, the content of monosaccharides and oligosaccharides in SPGT was notably increased (Figure 3E). Analysis of SGT and PGT revealed that shaking and piling treatments elevated the levels of various sugars in SPGT. For instance, trehalose, raffinose, sucrose, and L-galactose apparently increased in shaken SGT, whereas palatinose and cellobiose showed more pronounced increases in piled PGT. This could be attributed to enzymatic and non-enzymatic effects during shaking and piling, respectively [36,37]. In summary, the increase in soluble sugars under the combined process of shaking and piling was significantly higher than that achieved by either process alone.

#### 3.2.6. Peptides and Nucleotides

In this study, 12 peptides and 10 nucleotides were detected (Figure 3F). Small molecular peptides in tea were often associated with the kokumi taste, particularly gamma-Glu-Gln and glutathione, which were considered the main contributors to the kokumi of green tea [38]. We found that gamma-Glu-Gln and glutathione were increased in PGT and SPGT. Therefore, the increase in kokumi in SPGT could be attributed to the piling treatment. In addition, besides amino acids, several nucleotides and their derivatives (such as adenosine and guanine) also possessed umami characteristics [39]. Compared to SGT and PGT, the nucleotide contents in SPGT exhibited different changes. This indicated that the change of nucleotides in SPGT was a result of the combined effect of the shaking and piling treatments.

In summary, the improvement of the taste quality in SPGT was closely related to the reduction in bitter and astringent compounds such as ester-catechins, flavonoid glycosides, and procyanidins, as well as the increase in soluble sugars and sweet-tasting amino acids. The combined effect of the shaking and piling treatments on the metabolites of summer green tea was not only reflected in the enhancement of individual treatment but also included a mutual offsetting effect. Furthermore, the sensory characteristics of tea were not directly related to changes in the concentration of any single component but rather depended on the coordinated and integrated interactions among multiple flavor compounds.

### 3.3. Characterization of Significant Changed NVMs Among GT, SGT, PGT, and SPGT

**Figure 4:**
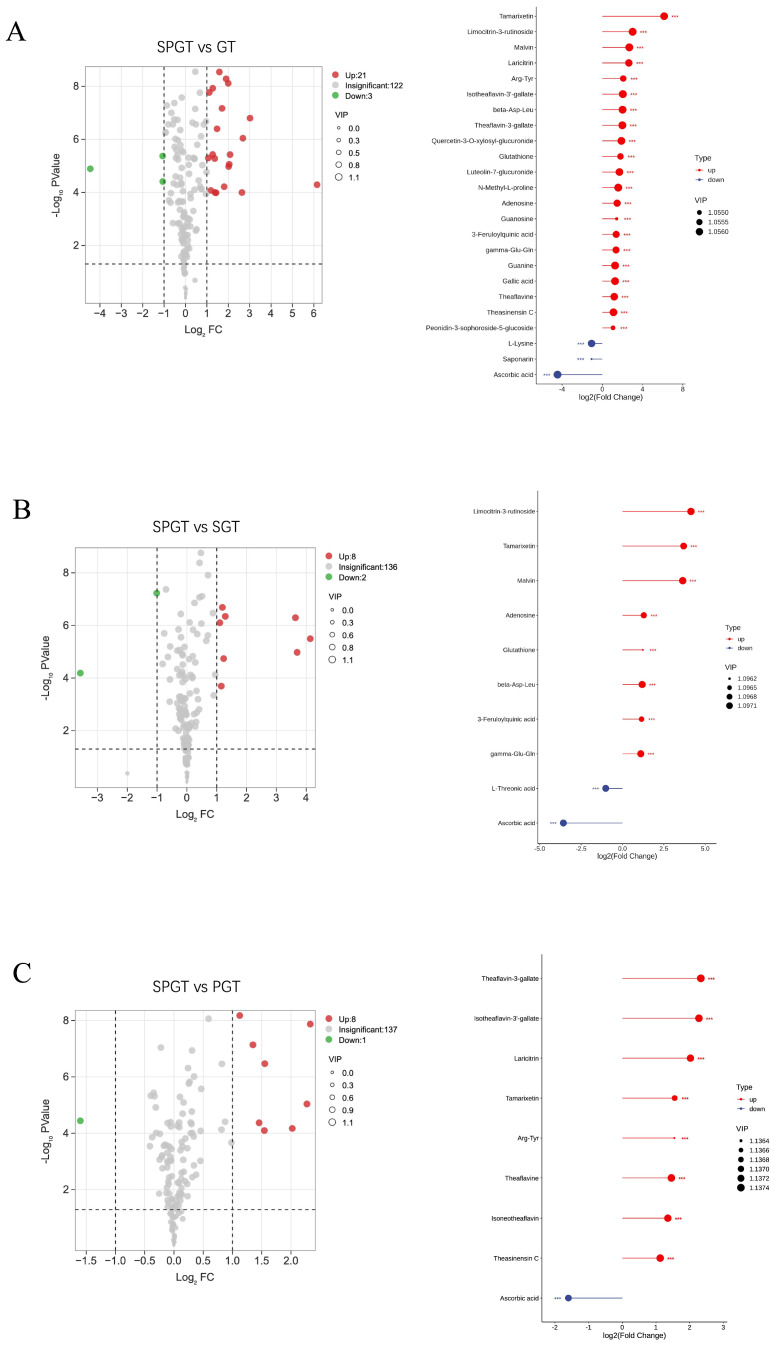
Volcano plots and differential NVMs in GT, SGT, PGT, and SPGT. (**A**) SPGT versus GT. (**B**) SPGT versus SGT. (**C**) SPGT versus PGT. ‘***’ indicated *p* < 0.001.

To better understand the influence of the combined shaking and piling process on NVM profiles, the differential metabolites in SPGT were determined by variable importance in the project (VIP) ≥1 and fold change ≥2 or ≤0.5, compared to GT, SGT, and PGT, respectively. As shown in Figure 4, the volcano plot provided a visual representation of the up-regulation and down-regulation of these differential metabolites.

Figure 4A revealed that 21 differential metabolites were significantly up-regulated (*p* < 0.001) in SPGT compared to GT, including theaflavins, glutathione, adenosine, guanine, and gamma-Glu-Gln, which contributed to the umami and kokumi tastes of green tea infusions. Additionally, three differential metabolites were significantly down-regulated (*p* < 0.001), namely L-lysine, saponarin, and ascorbic acid. Notably, the majority of NVMs in SPGT were up-regulated, suggesting that the transformation and generation of non-volatile compounds dominated the integrated shaking and piling process. When compared to SGT and PGT, the number of differential metabolites identified in SPGT was 10 (8 up-regulated, 2 down-regulated) and 9 (8 up-regulated, 1 down-regulated), respectively (Figure 4B,C). Our results indicated that the piling treatment increased the levels of glutathione, adenosine, 3-feruloylquinic acid, malvin, limocitrin-3-rutinoside, beta-Asp-Leu, and gamma-Glu-Gln (*p* < 0.001). Conversely, shaking treatment elevated the levels of isoneotheaflavin, theaflavine, isotheaflavin-3′-gallate, theaflavin-3-gallate, laricitrin, theasinensin C, and Arg-Tyr (*p* < 0.001). These variations in metabolite types could be attributed to the specific processes of shaking and piling. Furthermore, ascorbic acid, a potent antioxidant, exhibited a significant decrease in SPGT (*p* < 0.001), indicating that intense oxidation reactions occurred during both the shaking and piling processes.

#### 3.3.1. Taste-Related Differential NVMs

**Figure 5:**
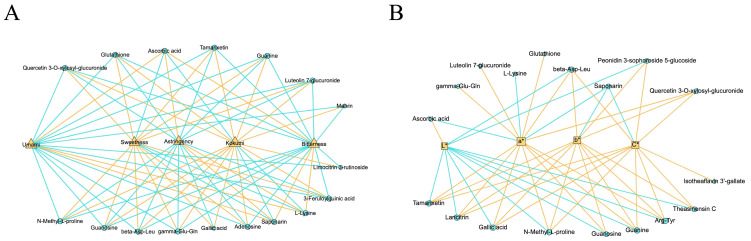
Correlation analysis of differential NVMs with quality attributes in GT, SGT, PGT, and SPGT. (**A**) Taste-related differential NVMs. (**B**) Color-related differential NVMs.

A correlation analysis was carried out between the differential metabolites of four summer green teas and taste attributes. As illustrated in Figure 5A, 17 differential metabolites showed a significant correlation with these tastes (|correlation| > 0.8, *p* < 0.05), including two amino acids and derivatives, six flavonoids and their glycosides, three organic acids, three peptides, and three nucleotides. The findings indicated that umami, bitterness, and astringency displayed significant positive correlations with ascorbic acid, L-lysine, and saponarin, respectively (*p* < 0.05). In contrast, sweetness and kokumi were negatively correlated solely with these three substances (*p* < 0.05). Furthermore, the results unveiled the presence of synergistic interactions among these differential substances in terms of taste attributes. For example, gamma-Glu-Gln and glutathione possessed kokumi attributes [38] and exhibited a promoting effect on sweetness. Conversely, several substances that enhanced umami, astringency, and bitterness showed corresponding inhibitory effects on sweetness and kokumi. Overall, the differential NVMs predominantly exhibited a positive influence on sweetness and kokumi tastes, concurrently exerting a negative influence on umami, bitterness, and astringency tastes during the integrated shaking and piling process in summer green tea.

#### 3.3.2. Color-Related Differential NVMs

A correlation analysis was carried out between the differential metabolites of four summer green teas and color parameters (L*, a*, b*, and C*). As illustrated in Figure 5B, 18 differential metabolites showed a significant correlation with these tastes (|correlation| > 0.8, *p* < 0.05), including two amino acids and derivatives, six flavonoids and their glycosides, two organic acids, four peptides, two nucleotides, one catechin, and one pigment. Most compounds exhibited a significant negative correlation with L* (*p* < 0.05), except for ascorbic acid. Research has indicated that as ascorbic acid undergoes oxidation, its color transitions from bright to dark [40]. Furthermore, most compounds exhibited a significant positive correlation with a*, b*, and C* (*p* < 0.05). Notably, only saponarin, identified as a blue-colored contributor in plants [41], displayed a significant negative correlation with b* and C* (*p* < 0.05). Overall, the differential NVMs predominantly exhibited a positive influence on yellowness and saturation, concurrently exerting a negative influence on brightness during the integrated shaking and piling process in summer green tea.

The integrated shaking and piling processing significantly modulated the sensory and chromatic attributes of summer green tea. These results mechanistically demonstrate that mechanical–thermal synergism in shaking and piling processing selectively remodels metabolite networks to enhance desirable sensory qualities and mitigate bitterness/astringency, providing a molecular foundation for the precision optimization of summer green tea.

### 3.4. Dynamic Changes in NVMs During Integrated Shaking and Piling Process

**Figure 6:**
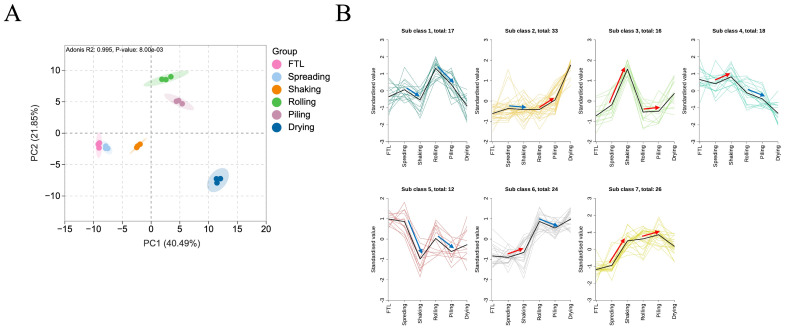
Dynamic changes in NVMs during the integrated shaking and piling process. (**A**) PCA score plot. (**B**) K-means plot. Arrows with blue and red color represent down-regulated and up-regulated, respectively.

The PCA diagram was performed to visually demonstrate the dynamic changes in NVMs during the integrated shaking and piling process. As depicted in Figure 6A, the first two principal components explained 40.49% and 21.85% of the total variation, respectively, with a combined contribution of 62.34%. The processed tea samples were clearly categorized (*p* < 0.05), exhibiting distinct trends in spreading, shaking, rolling, and piling. The findings indicate that the integrated shaking and piling process had a notable influence on the changes in NVMs. Furthermore, seven distinct change tendencies of NVMs were observed during the integrated shaking and piling process (Figure 6B and Table S1). Among these, 84 metabolites exhibited an increasing trend in content during shaking, while 62 metabolites showed a decreasing trend. Meanwhile, during piling, 75 metabolites demonstrated an increasing trend, and 71 metabolites exhibited a decreasing trend. These results suggest that the transformation and generation of NVMs were the predominant trends during the integrated shaking and piling process. Additionally, the different change tendencies of NVMs between shaking and piling demonstrate the distinct influence mechanisms of these two methods. These findings validate the above speculation concerning the differences in NVMs among the four summer green tea samples.

## 4. Conclusions

In this study, we conducted an evaluation of the taste and color attributes of summer green tea subjected to an integrated shaking and piling treatment. The combined treatment significantly enhanced the sweetness and kokumi intensity while reducing umami, astringency, and bitterness. Colorimetric analysis revealed an increase in yellowness and improvement in color saturation compared to conventional processing. The NVMs were profiled using UPLC-Triple-TOF-MS, identifying 146 compounds with marked taste-aligned variations. Based on a variable importance in the project (VIP) ≥ 1 and fold change ≥2 or ≤0.5, 21 differential NVMs were selected, showing significant accumulation in SPGT. These observations hinted at the dominance of non-volatile compound transformations and generations during the integrated shaking and piling process. Correlation analysis between these differential NVMs and taste/color attributes pointed to their beneficial effects on sweetness, kokumi flavors, yellowness, and color saturation. Subsequently, the dynamic alterations in NVMs were confirmed, further substantiating the occurrence of non-volatile compound transformations and generations throughout the integrated shaking and piling process.

In conclusion, our research demonstrates that integrated shaking and piling processing provides a transformative strategy for summer green tea valorization, achieving the dual objectives of quality enhancement and resource efficiency. By elucidating the metabolite remodeling mechanisms underlying sensory-chromatic improvements, our approach enabled the conversion of underutilized summer tea leaves into premium-grade products, projected to reduce seasonal agricultural waste while increasing market value in major production regions.

## Data Availability

The data presented in this study are available on request from the corresponding author.

## Supplementary Materials

The following supporting information can be downloaded at https://www.mdpi.com/article/10.3390/foods14071284/s1, Table S1 List of the 146 compounds of each samples using non-targeted metabolomics analysis.
